# Spatial distribution patterns and risk factors of hookworm disease in China: A study based on successive national surveillance

**DOI:** 10.1371/journal.pntd.0013526

**Published:** 2025-09-30

**Authors:** Huihui Zhu, Jilei Huang, Jinxin Zheng, Changhai Zhou, Tingjun Zhu, Mizhen Zhang, Luyuan Zhao, Xiaohong Wu, Jingbo Xue, Xiao-Nong Zhou, Shizhu Li, Menbao Qian

**Affiliations:** 1 National Institute of Parasitic Diseases, Chinese Center for Disease Control and Prevention, Shanghai, China; 2 Chinese Center for Tropical Diseases Research, Shanghai, China; 3 National Key Laboratory of Intelligent Tracking and Forecasting for Infectious Diseases, Shanghai, China; 4 Key Laboratory on Parasite and Vector Biology, Ministry of Health, Shanghai, China; 5 WHO Centre for Tropical Diseases, Shanghai, China; 6 National Center for International Research on Tropical Diseases, Ministry of Science and Technology, Shanghai, China; 7 School of Global Health, Chinese Center for Tropical Diseases Research-Shanghai Jiao Tong University School of Medicine, Shanghai, China; 8 Sichuan Center for Disease Control and Prevention, Chengdu, Sichuan, China; Seoul National University College of Medicine, KOREA, REPUBLIC OF

## Abstract

**Background:**

Hookworm infection, a neglected tropical disease (NTD) causing iron-deficiency anaemia and malnutrition in low-income populations with poor sanitation, poses a considerable public health challenge in China and worldwide.

**Methods:**

National surveillance across 31 provincial-level administrative divisions (PLADs) from 2016 to 2021 assessed regional and population-specific hookworm prevalence. Geospatial methods, such as global and local autocorrelation, hotspot detection, spatiotemporal clustering detection and standard deviation ellipse (SDE) analysis characterized distribution patterns. Machine learning identified key determinants and their associations with infection rates, revealing primary influence factors based on 7,929 township records and 40 environmental, climatic and anthropogenic variables.

**Results:**

Significant geographic disparities emerged, with the highest infection rates in south-western regions and the lowest in the Northeast. Spatial analyses demonstrated significant clustering, with persistent south-western hotspots and north-eastern coldspots (*P* < 0.001). Spatiotemporal scanning identified three significant clusters, while SDE analysis indicated stable northeast-southwest orientation with minimal centroid variation. Females and individuals ≥60 years showed elevated susceptibility. Machine learning demonstrated strong predictive capacity, with key risk factors identified as the frequency of barefoot farming, land cover, average relative humidity in the third quarter and average monthly sunshine duration in the third quarter.

**Conclusions:**

Hookworm disease clusters in south-western China, disproportionately affecting women and the elderly. Barefoot farming emerged as the primary risk factor, with infection rates positively associated with temperature, humidity and negatively with sunlight duration. The results support recommendations to target intervention zones in endemic areas, implement population-specific prevention programs and intensify health education to advance transmission control.

## Author summary

Hookworm infection remains a serious health problem in China, especially in poor rural areas. Our national study (2016–2021) found the disease mainly affects southwest regions, with women and older adults being most vulnerable. Using mapping technology and computer analysis, we discovered that working barefoot in fields is the biggest risk, along with living in warm, humid areas with less sunlight. The disease shows clear patterns - staying concentrated in the southwest while rarely appearing in northeast China. These findings highlight three key solutions: (1) special attention on shoe-wearing in farm workers, (2) focusing control efforts on high-risk southwest regions, and (3) educating vulnerable groups. Our approach combining geographic mapping with artificial intelligence not only helps China fight hookworm but could also work for similar diseases in other countries.

## Introduction

Hookworm disease, classified as a soil-transmitted helminth infection, arises from the parasitisation of the human small intestine by generally two species: *Necator americanus* and *Ancylostoma duodenale* [1–3]. This infection induces primarily chronic, iron-deficiency anemia, which lead to fatigue and malnutrition, especially in children and pregnant women [4,5]. As a Neglected Tropical Disease (NTD), hookworm disease is identified as an important infectious disease requiring control within the framework of the 2030 Agenda for Sustainable Development (SDG) established by the United Nations [6–8]. It imposes a significant disease burden both in China and worldwide [2,5,9–13]. In spite of being preventable and treatable, it is estimated that approximately 1.3 billion individuals are afflicted globally, and hookworm infection accounts for approximately 65,000 deaths annually, contributing to 845,000 disability-adjusted life years (DALYs) and resulting in productivity losses ranging from 6.0% to 35.3% [14]. The latest National Survey in China, carried out in 2015, indicates an estimated 16.97 million infections corresponding to 2.6%. Among the provincial-level administrative divisions (PLADs) surveyed, Sichuan exhibited the highest infection rate at 14.6%, followed by Hainan and Chongqing at 8.1% and 5.7%, respectively [15].

Hookworm infection is a continuing significant public health challenge, underscoring the need for a more comprehensive understanding of its spatial distribution and associated risk factors to inform targeted interventions. Existing studies on the spatial distribution of hookworms in China have primarily focused on localized patterns, with no comprehensive national assessment conducted to date [16–17]. Previous research has identified various risk factors, including temperature, proximity to water sources, sanitation conditions, agricultural practices, etc [18–23]. However, these studies typically rely either on international datasets or localized field surveys that lack nationally representative data accurately reflecting the epidemiological context in China. Furthermore, they have not comprehensively integrated natural, social or demographic determinants within a unified analytical framework. This gap necessitates the application of new and effective methodologies alongside a nationally representative dataset. Machine learning (ML) offers distinct advantages in handling high-dimensional data and modelling nonlinear relationships, which would enable automated feature selection, leverage ensemble learning techniques and integrate multi-source datasets, making it particularly suitable for risk factor analysis. In 2016, China implemented a national surveillance system for hookworm infections, conducted annually across 200–450 counties in all available 31 PLADs [24–29]. This initiative together with public datasets provided a valuable opportunity to utilize ML methods to analyze the spatial distribution and risk factors associated with hookworm infection on a national scale.

This study aims to elucidate the spatial distribution patterns and risk factors associated with hookworm disease in China, thereby providing essential data and a scientific foundation for the effective control of this public health issue.

## Methods

### Ethical statement

This article was based on surveillance data from the National Institute of Parasitic Diseases (NIPD) at the Chinese Center for Disease Control and Prevention (China CDC). The study obtained approval from the Institutional Ethical Review Committee of NIPD, China CDC (document No. 2021006). No personal information was disclosed throughout the study. Oral informed consent for publication was obtained from all participants. For participants under 18 years of age, oral informed consent was obtained from their guardian.

### Data resources

#### Surveillance areas and population.

The national surveillance conducted from 2016 to 2021 encompassed 31 PLADs throughout the country. All PLADs adhered to the same annual surveillance plan, wherein surveillance counties were selected based on predetermined criteria [30]. A standardized sampling method was employed in each selected county, with a systematic division into five geographical areas: the east, west, south, north, and Centre. Subsequently, one township was randomly chosen from each area, followed by the selection of one village from each township, resulting in a total of five villages selected per county. Cluster sampling was utilized to select 200 permanent residents from each village, amounting to a total of 1000 individuals surveyed in each county. Fecal samples exceeding 30g were collected from the residents, and the Kato-Katz method was employed for fecal examination [31].

#### Map data.

The map layers depicting China’s administrative divisions were obtained from the official website of the Ministry of Civil Affairs of China (http://xzqh.mca.gov.cn/map), with the approval number GS (2022) 1873.

#### Possible risk factors of hookworm disease.

Meteorological, environmental and anthropogenic factors were collected from various sources. Climate factors were obtained from the Resource and Environmental Science Data Platform (https://www.resdc.cn/data.aspx?DATAID=349). Average values for each year and each quarter during this period were subsequently calculated. Most of the environmental factors were sourced from The NASA Shuttle Radar Topographic Mission (SRTM) (http://srtm.csi.cgiar.org/), while land cover was collected from national surveillance, geographic coordinates were exported from map data after they were matched with names of the surveillance sites. The anthropogenic factors were sourced from the Socioeconomic Data and Applications Center (SDAC) in the U.S. (https://sedac.ciesin.columbia.edu). Additional data related to human activities, such as the frequency of barefoot farming (Barefoot.farming), predominant local industries (Industry), annual per capita net income of residents (Income), gross domestic product (GDP), fraction of sanitary toilets of households in villages (Fr.sanitary.toilets) and primary drinking water sources (Drinking.water) were collected from national surveillance sites. These variables were listed and numbered as seen in Table 1.

**Table 1:** Variables used in the machine learning Model.

Variable number	Variables	Median	IQR/Category	Variable description
1-1	Pre	66.82	44.66-113.22	Annual average precipitation (mm)
1-2	Pre01	45.24	25.02-114.60	Average monthly precipitation in the first quarter (mm)
1-3	Pre02	144.39	104.77-200.61	Average monthly precipitation in the second quarter (mm)
1-4	Pre03	47.19	27.63-81.76	Average monthly precipitation in the third quarter (mm)
1-5	Pre04	13.02	4.43-40.72	Average monthly precipitation in the fourth quarter (mm)
1-6	Rhu	70.72	59.55-78.58	Annual average relative humidity (%)
1-7	Rhu01	62.81	48.57-77.24	Average relative humidity in the first quarter (%)
1-8	Rhu02	76.45	69.18-80.47	Average relative humidity in the second quarter (%)
1-9	Rhu03	73	64.76-79.56	Average relative humidity in the third quarter (%)
1-10	Rhu04	69.79	56.41-77.79	Average relative humidity in the fourth quarter (%)
1-11	Ssd	166.43	129.74-207.97	Annual sunshine duration (hours)
1-12	Ssd01	197.93	126.93-244.31	Average monthly sunshine duration in the first quarter (hours)
1-13	Ssd02	193.32	166.11-219.53	Average monthly sunshine duration in the second quarter (hours)
1-14	Ssd03	158.33	122.43-195.36	Average monthly sunshine duration in the third quarter (hours)
1-15	Ssd04	134.46	86.11-184.62	Average monthly sunshine duration in the fourth quarter (hours)
1-16	Tem	15.39	10.199 -17.955	Annual average temperature (°C)
1-17	Tem01	16.14	12.13-18.11	Average temperature in the first quarter (°C)
1-18	Tem02	26.27	22.91-27.59	Average temperature in the second quarter (°C)
1-19	Tem03	15.69	10.111 -18.663	Average temperature in the third quarter (°C)
1-20	Tem04	3.25	3.726-7.939	Average temperature in the fourth quarter (°C)
2-1	NDVI	0.36	0.22-0.54	Normalized Difference Vegetation Index
2-2	Waterdistance	3628.46	1782.86-6576.01	Distance to the nearest waterbody
2-3	Slope	1.13	0.25-4.77	Local slope
2-4	Elv	198	57.00-739.00	Elevation
2-5	Landcover	12	9.00-12.00	Vegetation Coverage
2-6	Soilmoisture	88.96	37.99-115.26	Moisture of soil
2-7	Landform	Categorical variable	Plateau, Mountain, Basin, Hill, Plain	Local landform
2-8	Lat	33.42	28.08-33.69	Latitude(°)
2-9	Lon	113.81	107.63-117.59	Longitude(°)
3-1	HII	26	18.00-30.00	Human Impact Index
3-2	HFP	40	28.00-46.00	Human Footprint Index
3-3	Barefoot.farming	Categorical variable	Often, Occasionally, Never	Frequency of barefoot farming
3-4	Industry	Categorical variable	Industry and Commerce, Agriculture, Fishery, Forestry, Animal Husbandry	Predominant local industries
3-5	Income	9500	5500-11911	Annual Per Capita Net Income of Household (Yuan)
3-6	Fr.sanitary.toilets	63.24	1.76-99.56	Fraction of Sanitary Toilets
3-7	Drinking.water	Categorical variable	Tap Water, Well Water, Pond Water, River and Lake Water, Other	Types of local drinking water
3-8	GDP	915.3	334.30-2567.40	Gross Domestic Product
3-9	POPdensity	308.6	109.00-790.00	Population density
3-10	Year	Categorical variable	2016, 2017, 2018, 2019, 2020	Year of hookworm surveillance

### Database construction

County-level infection indicators for hookworm from 2016 to 2021, comprising the number of individuals investigated, the number of individuals infected, and the infection rate, were computed. ArcGIS 10.2 software was employed to align the county-level database with the geographical coordinates of each county, creating a spatial analysis database.

**Fig 1:**
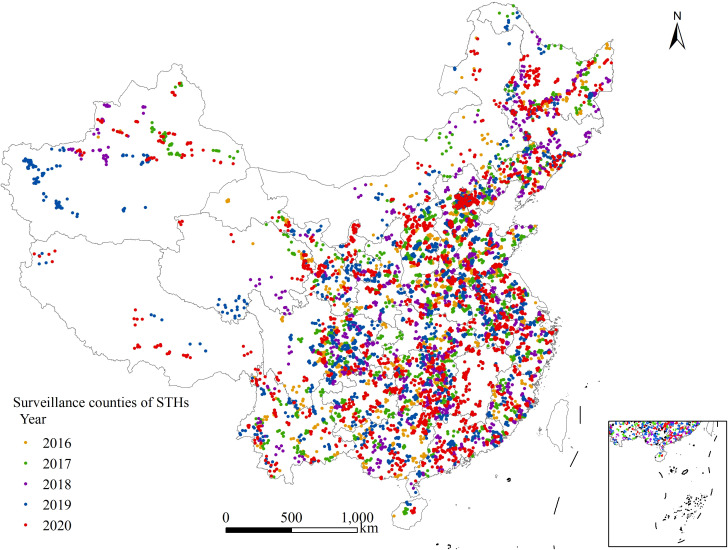
Distribution of national surveillance sites for STHs from 2016 to 2020.

The machining learning database comprised 7,929 township records and 40 variables, with the townships sourced from 31 PLADs across China. The distribution of the surveillance counties in which these townships are located is illustrated in Fig 1. Detailed information regarding the variables is provided in Table 1.

### Data analysis

#### Endemic status of hookworm disease in China.

The database of all 31 PLADs was analyzed using SAS 9.3 (Version 70068130) software. The hookworm infection rate was calculated as the number of positive cases divided by the total number of individuals tested. The annual infection rates of hookworm were calculated, along with the infection rates categorized by PLADs, gender and age groups. Statistical comparisons of infection rates were conducted using the Chi-square (χ^2^) test, with a significance level set at α < 0.05.

#### Spatial analysis.

County-level infection indicators for hookworm from 2016 to 2021, comprising the number of individuals investigated, the number of individuals infected, and the infection rate, were computed. ArcGIS 10.2 software was employed to align the county-level database with the geographical coordinates of each county, creating a spatial analysis database. Subsequently, the distribution patterns of hookworm infection in China were characterized by these following methods:

1 Global Spatial Autocorrelation Analysis

The overall spatial dependence of hookworm prevalence using Global *Moran’s I* statistic. This measure quantifies whether the infection rates exhibited a clustered (*I* > 0), dispersed (*I* < 0), or random (*I* ≈ 0) spatial pattern across the study region. Statistical significance (*P* < 0.05) was determined through 999 Monte Carlo permutations to evaluate whether the observed spatial pattern occurred by chance.

2 Hotspot Analysis (Getis-Ord Gi*)

Local spatial clusters of high and low infection rates were identified using the Getis-Ord Gi* statistic. This localized approach detected statistically significant (*P* < 0.05, with False Discovery Rate correction) hotspots (areas with unusually high prevalence) and coldspots (areas with unusually low prevalence) while accounting for spatial weighting through a distance-based neighborhood matrix.

3 Spatiotemporal Cluster Analysis

Space-time scan statistics (Kulldorff’s method) were implemented to detect significant spatiotemporal clusters of hookworm infection. The analysis employed a cylindrical scanning window that moved across both space and time. Likelihood ratio tests with 999 Monte Carlo simulations identified clusters where observed cases significantly exceeded expected counts (*P* < 0.05).

4 Standard Deviation Ellipse (SDE) Analysis

This method provided directional trends and dispersion dynamics of hookworm distribution were by the generation of ellipses based on the standard deviations (SDs) of x- and y-coordinates of infection cases, revealing: 1) the spatial centroid of disease distribution, 2) the orientation of the primary axis of spread, and 3) the degree of directional bias in transmission patterns across different time periods.

#### Machine learning (ML).

Town-level infection rates of hookworm from 2016 to 2020 were calculated using the national surveillance database, with data mapped according to the longitude and latitude of each township. These infection rates were then matched with corresponding environmental, meteorological, and human variables so that the database was constructed.

R software (4.3.1) was then used to analyze the influencing factors of hookworm disease.

Based on the constructed database, Variable selection was performed using a cross-validated recursive feature elimination (RFE) algorithm. In this analysis, the infection rate of hookworm served as the dependent variable, with the environmental, meteorological, and human variables as independent ones. Specifically, during each resampling iteration, the dataset was first split into 70% training and 30% validation sets for each model, followed by recalculation and ranking of feature importance. For each candidate feature subset size S_i_ (where i = 1,2,3...n), the following procedure was repeated: the top S_i_ most important features were selected, a model was retrained using this subset and evaluated on the validation set, and feature importance recalculated and re-ranked. The validation performance across different subset sizes (S_i_) was then systematically compared to determine the optimal number of features. The final feature set was estimated and used to build the ultimate model on the complete training dataset. All analyses were implemented using the Caret package in R software.

After the variables had been selected, ML models were constructed, including the following models: linear regression model (LM), random forest model (RF), gradient boosting machine (GBM), and extreme gradient boosting (XGBoost). The analytical procedure was started by randomly partitioning the study database into a training set (70% of the data) and a testing set (30%). The training set was then divided into 5 cross-validation subsets, where the models were iteratively trained on 4 subsets and validated on the remaining subset to optimize model parameters. The resulting model was subsequently used to evaluate performance on both the training set and the testing set. The optimal model was selected based on performance metrics, followed by ranking of variable importance and analysis of inter-variable dependencies within the model. All modeling procedures were implemented using the Caret package in R software. The performance was evaluated by comparing the mean absolute error (MAE), root mean square error (RMSE), and R², coefficient of determination values across candidate models, with the optimal model selected based on smaller MAE and RMSE values coupled with a relatively larger R² values. The RMSE and MAE metrics were computed using the Metrics package in R software, while the R² values were calculated using the Ggpubr package.

Further analysis was performed based on the optimal model selected, where the importance of the variables was ranked, and partial dependence graphs (PDPs) were created. The relative importance of feature variables was determined by quantifying the reduction in RMSE following the inclusion of each individual variable into the model, with greater RMSE reduction indicating higher variable importance. Based on the optimal ML model previously established, the RMSE reduction attributable to each variable and visualized, the results were calculated and demonstrated by bar plots. This analysis was implemented using the Featureeffect function from the Iml package in R software, with subsequent graphical representation generated through the plot function in the base Graphics package. The dependency relationship between variables was examined by assessing how the predicted outcome (Y) changes when varying a specific predictor variable while holding all other independent variables (X) at their mean values. In machine learning models, these relationships were visualized through PDPs, implemented using the partial function from the Pdp package in R software.

## Results

### Epidemic status

#### Total infection rate.

Between 2016 and 2021, the hookworm infection rates were 3,515 out of 262,380 (1.3%), 2,974 out of 29,778 (1.0%), 2,911 out of 326,207 (0.9%), 3,580 out of 424,766 (0.8), 2,106 out of 415,672 (0.5%) and 3,856 out of 424,303 (0.7%), respectively. Over the period, the infection rates of hookworms exhibited a general decrease trend from 2016 to 2020, and the reduction rate was 61.9%, moreover, there was a slight increase from 2020 to 2021, with the increase rate of 31.4%.

#### Regional distribution.

In 2016, the infection rates exceeded 1.0% in 8 PLADs, with Sichuan exhibiting the highest infection rate (8.2%), followed by Hainan (7.2%), Chongqing (6.4%), Yunnan(5.2%), Guizhou (2.9%), Guangxi (2.8%), Fujian (2.7%),Hunan (2.0%) and Zhejiang (1.1%). In the remaining 22 PLADs, infection rates were all below 1.0%, with no cases of hookworm infection reported in 10 PLADs (in Xizang, surveillance was only conducted during 2019–2021).

In 2021, the infection rates exceeded 1.0% in 6 PLADs, with Yunnan exhibiting the highest infection rate (4.9%), followed by Sichuan (4.7%), Chongqing (2.6%), Zhejiang (2.2%), Hainan (2.1%), and Fujian (1.1%). In the remaining 25 PLADs, infection rates were all below 1.00%, with no cases of hookworm infection reported in 16 PLADs.

**Fig 2:**
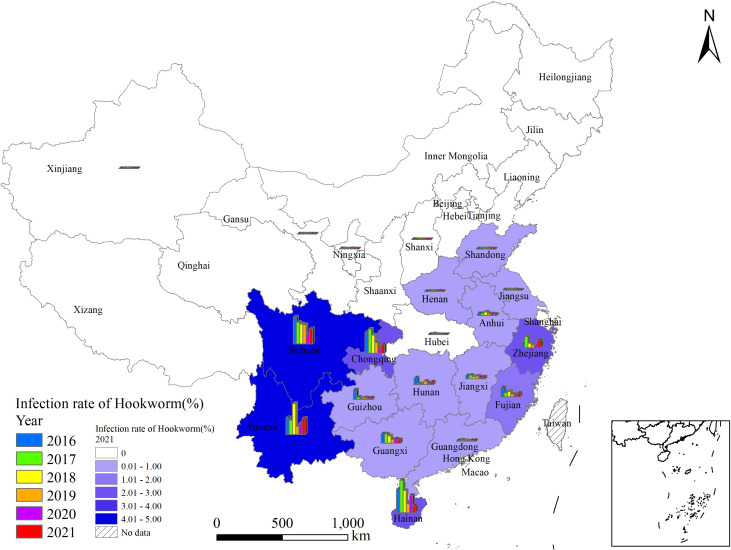
Geographic distribution of Hookworm at national surveillance sites from 2016 to 2021.

From 2016 to 2021, the hookworm infection rates were relatively high in four PLADs in China, namely Sichuan, Yunnan, Chongqing, and Hainan, and the infection rates has decreased by 41.2%, 12.9%, 20.3%, and 33.0%, respectively in these four PLADs between 2016 and 2021. During these years, the number of PLADs without hookworm infections increased to 10, namely Beijing, Tianjin, Hebei, Inner Mongolia, Liaoning, Jilin, Heilongjiang, Shanghai, Shaanxi, and Qinghai. Moreover, Shanxi, Xinjiang and Gansu recorded infection rates below 0.1%, with the years of cases limited to 2016. The remaining provinces demonstrated a general pattern of fluctuating decline in infection rates (Fig 2).

#### Gender distribution.

Between 2016 and 2021, the infection rates of hookworm in male were 1.2%, 0.9%, 0.8%, 0.8%, 0.4% and 0.6%, respectively, with the corresponding data in females 1.5%, 1.1%, 1.0%, 0.9%, 0.6% and 0.7%. The infection rate exhibited a general pattern of fluctuating decline in both males and females, with females consistently showing higher infection rates compared to males throughout the study period (*P* < 0.001).

#### Age distribution.

Between 2016 and 2021, the infection rate of hookworm was most pronounced among individuals aged ≥60 years and older, followed by those in the age ranges of 45–59, 15–44 and 7–14 years in that order. Conversely, the lowest rates of infection were observed in the 0–6 age group. Notably, there were statistical differences in hookworm infection rates across different age cohorts during this time period (*P* < 0.001). Overall, there was a fluctuating downward trajectory in the infection rate of hookworm across all age groups.

### Spatial analysis

#### Global spatial autocorrelation analysis.

The findings of the global autocorrelation analysis revealed *Moran’s I* values for the distribution of hookworm disease in China between 2016 and 2021 consistently exceeded 0, with corresponding *P*-values consistently below 0.0001. These results suggest a spatially positive correlation in the distribution of hookworm disease across China over the six-year period investigation, as summarized in Table 2.

**Table 2:** Global spatial autocorrelation analysis of Hookworm disease in China from 2016 to 2021.

Year	Moran ‘s *I* value	Expected Moran’s *I* value	Variance	*Z* value	*P* value
2016	0.317643	-0.003922	0.000617	12.949817	0.000000
2017	0.153637	-0.007092	0.000498	7.200960	0.000000
2018	0.383730	-0.003135	0.000219	26.118805	0.000000
2019	0.243657	-0.003096	0.000330	13.577012	0.000000
2020	0.183639	-0.002674	0.000117	17.253500	0.000000
2021	0.172327	-0.003546	0.000310	9.988449	0.000000

The absolute value of *Moran’s I* indicates the strength of spatial autocorrelation, with larger values signifying stronger spatial clustering. Based on the values of Moran’s I, the years with the strongest spatial clustering were found to be 2018, 2016 and 2019. The variance measures the dispersion of the estimated *Moran’s I* values; a smaller variance indicates greater stability in the estimates. The variance values for the spatial autocorrelation analysis from 2016 to 2021 were all < 0.001 suggesting that the estimates are stable (Table 2).

#### Hotspot analysis.

**Fig 3:**
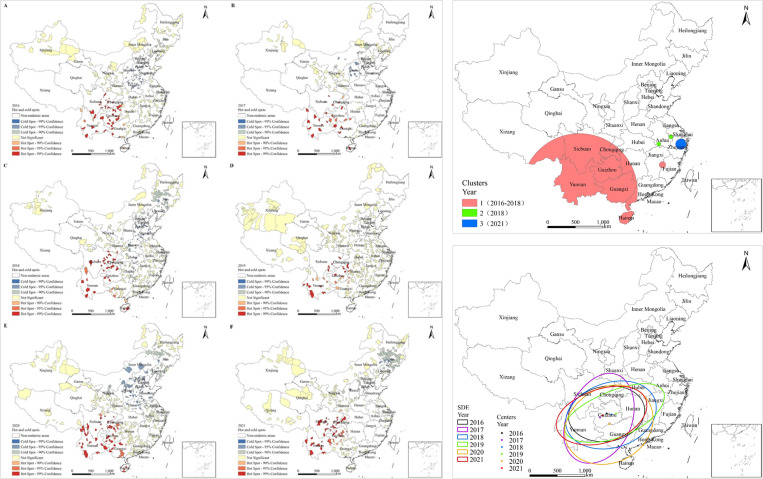
Results of spatial distribution analysis of Hookworm disease in China from 2016 to 2020. (Left figure: Hotspot analysis, A-F were Hotspot analysis of Hookworm disease from 2016 to 2021 respectively; Upper-right figure: Space-time scanning analysis; Lower-right figure: Standard deviation ellipse analysis).

Hotspot analysis conducted for the period spanning from 2016 to 2021 revealed localized spatial clustering in the distribution of hookworm infection in China. The highest concentrations of hotspots were predominantly deen in the southwestern regions of China, particularly in Sichuan, Yunnan, Chongqing, Guizhou, and Hainan. Among the five PLADs representing Sichuan, Yunnan, Chongqing, Guizhou, and Guangxi consistently exhibited hotspots over the past six years, whereas Hainan displayed hotspots in 2016, 2017, 2018 and 2020, with none identified in 2019 and 2021. The cold spots were predominantly located in north-eastern China. Notably, but neither coldspots nor hotspots were found in PLADs representing Xinjiang, Xizang, Qinghai, Gansu, Ningxia, Fujian, Guangdong, and Zhejiang, which are located in the northwest or southeast regions of China (Fig 3).

#### Spatiotemporal clustering analysis.

The retrospective spatiotemporal scanning analysis revealed the presence of three regions with hookworm disease clusters between 2016 and 2021 two of which identified from 2016 to 2018 ad a single one observed in 2021. These findings indicate the existence of spatiotemporal clustering in the distribution of hookworm disease during this time period. The predominant cluster area during the period from 2016 to 2018 was situated in China’s Southwest encompassing PLADs representing the provinces Yunnan, Sichuan, Chongqing, Guizhou, Hainan, Guangxi, Guangdong and Hunan. This cluster had a radius of 1,010.4 km centred over longitude 102.01°E and latitude 23.58°N. Its relative risk (RR) indicated that the risk of hookworm infection was 7.55 times higher than in the surrounding regions, and the log likelihood ratio (LLR) of 6454.08 (*P* < 0.000) confirmed the statistically significant spatiotemporal aggregation of hookworm cases in this area. The remaining aggregations were relatively small, located in Jiangxi and Fujian provinces and with additional aggregation regions from 2016 to 2018. Furthermore, in 2018, there were two aggregation zones situated in the southern and eastern parts of Anhui Province and another such located was located in Zhejiang Province in 2021 (Fig 3).

#### Standard deviation ellipse analysis (SDE).

The centroid of the ellipse was consistently situated in Guizhou, except for 2020 when it was located in Guangxi. The ellipse located in the south-western region, encompassed PLADs representing Sichuan, Yunnan, Guizhou, Chongqing, Guangdong, Guangxi, Hainan, Jiangxi and Hubei. It reflected the weighted mean centre of the spatial distribution, serving as a key indicator of the core concentration area. The primary concentration area of hookworm distribution was Guizhou. Between 2016 and 2021, the spatial distribution of hookworm disease exhibited a SDE rotation angle ranging from 32.64 to 83.87, with the rotation angle at its minimum in 2017 and peak in 2016. The rotation angle is defined as the clockwise angle from true north, and characterizes the dominant directional trend of the spatial pattern. The rotation angles were all clockwise angle from true north <90, which is a predominant northeast-southwest orientation. The ratio of the major to minor axes of the ellipse ranged from 1.31 to 2.51. The ratio quantifies the directionality strength of the distribution—a higher ratio indicates a more linear/spatially elongated pattern. The distribution of hookworm was a moderate directionality (obvious oval) within the ration range (Fig 3).

Between 2016 and 2021, the displacement of the centre points did not exhibit significant magnitude. When comparing with the movement in 2016, it was observed that in 2017 and 2021, the centre point of the ellipse shifted towards the south-western direction, whereas in 2018, 2019, and 2020, the movement was towards the southeast direction indicating a minor overall migration trend (Fig 3).

### Machine learning (ML)

#### Database and variable selection.

**Fig 4:**
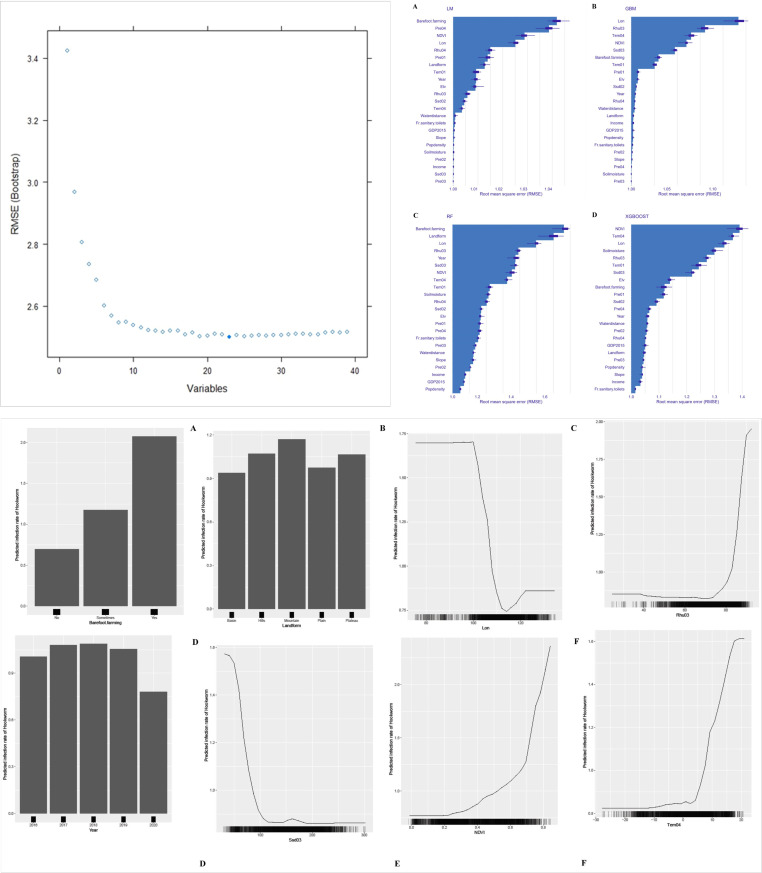
Results of machine learning. (Upper-left figure: variables selections by REF (A: LM model; B: GBM model; C: RF model; D: XGBOOST model); Upper-right figure: Variable importance in each model (A: Barefoot.farming; B: Landform; C: Longitude; D: Rhu03; E: Year; F: Ssd03; G:NDVI; H: Tem04); Lower figure: The partial Dependence Plot between estimated infection rate of Hookworm and important variables in RF model).

The recursive feature elimination (RFE) algorithm indicated that the model achieved the highest accuracy when 23 variables were included (Fig 4). Consequently, these 23 variables were incorporated into the model.

#### Model construction.

The fitting performance of the four models was compared, with the results presented in Table 3. It is evident that the RF model exhibited the highest coefficient of determination (R²) and the lowest values for both Mean Absolute Error (MAE) and Root Mean Square Error (RMSE), indicating that the RF model is the optimal choice among the evaluated models.

**Table 3:** Fit parameters of model performance in the training set and testing set.

Model	Training Set			Testing Set		
	RMSE	MAE	R^2^	RMSE	MAE	R^2^
RF model	1.27	0.46	0.9	2.65	0.89	0.5
XGBOOST model	1.51	0.67	0.79	2.79	0.98	0.42
LM model	2.99	1.44	0.17	3.35	1.49	0.18
GBM model	2.47	0.98	0.45	3.02	1.06	0.34

RMSE = root mean square error; MAE = mean absolute error;the R² = coefficient of determination RF = random forest; XGBoost = extreme gradient boosting; LR = linear regression; GBM = gradient boosting machine.

#### Variable importance analysis and PDP plot.

The importance sequence diagram for the variables in the RF model indicated that the top eight variables, ranked according to importance, were as follows: Barefoot farming, landform, longitude, average relative humidity in the third quarter (Rhu03), year, average sunshine duration in the third quarter (Ssd03), NDVI, and average temperature in the fourth quarter (Tem04) (Fig 4).

The PDP was generated based on the RF model (Fig 4). It revealed that, among the categorical variables, “barefoot farming” presents significant hookworm risk factors when compared to the “never” category. Specifically, the RR values for “sometimes” and “often” were 1.62 and 3.97, respectively, with a significance level of *P* < 0.0001. Regarding “Landcover,” both “mountainous areas,” “plateaus,” and “hills” were identified as risk factors in comparison to “plains,” with RR values of 1.21, 1.09, and 1.10, respectively, and a significance level of *P* < 0.05. Additionally, the analysis indicated that the hookworm infection rates of from 2016 to 2019 exhibited statistically significant differences when compared to the year 2020, with RR values of 1.25, 1.35, 1.36 and 1.31, respectively, all at *P* < 0.0001.

In the analysis of continuous variables, it was observed that there was no significant variation in the hookworm infection rate for longitudes less than 100° or greater than 122°. However, within the longitude range of 100° to 113°, the infection rate of hookworms decreased with increase of longitude. Conversely, between 114° and 122°, there was a slight increase in the infection rate with increasing longitude. Regarding the variable Rhu03, when its value was < 73, the hookworm infection rate also remained very low and did not exhibit significant variation with changes in Rhu03. In contrast, when Rhu03 was between 73 and 93, there was a significant increase in the infection rate as Rhu03 increase. For the variable Ssd03, when its value was below 130 hours, the hookworm infection rate significantly decreased with increasing Ssd03. However, within the range of 130–300 hours, the infection rate remained very low, showing no significant change with variations in Ssd03.

With regard to NDVI, it was found that values <0.2 coincided with very low hookworm infection rates but did not exhibit significant changes with variations in NDVI. However, within the range of NDVI from 0.2 to 0.85, the infection rate of hookworm incidence increased with rising NDVI values. Similarly, for the variable Tem04, when values were within the range of -30°C to 15°C, the hookworm infection rate was also very low and did not show significant changes with fluctuations in Tem04. Conversely, in the temperature range of 15°C to 22°C, the infection rate of hookworm increased with rising temperatures.

## Discussion

To understand the epidemiological status and trends of this parasitic infections, the national surveillance of hookworm disease in China represents the most comprehensive and systematic approach, beyond nationwide surveys. The national surveillance revealed that from 2016 to 2021, there was a general pattern of fluctuating decline in infection rates of hookworm, with a concomitant contraction in its endemic range. This could be attributed to sustained surveillance systems, implementation of targeted control measures, concomitant economic growth, and subsequent elevation of living standards. The distribution of hookworm disease exhibited distinct regional and demographic characteristics. In terms of regional distribution, spatial analysis confirmed clustered distribution of hookworm disease, with significant hotspots in the southwest and cold spots in the northeast, with a minor over migration trend. The south-western regions characterized by high temperatures and humidity provide favorable conditions for the survival of hookworm. Additionally, the relatively underdeveloped economy in these areas, coupled with agricultural practices in rural settings, facilitates the transmission of hookworm [32]. As such, future prevention and control efforts should prioritize these regions, with the establishment of pilot initiatives in high-prevalence areas. Conversely, the climate in northeast was not suitable for hookworm survival. Despite these variations, it is imperative that vigilance in prevention and control measures is maintained across all regions. Regular surveillance and timely implementation of control measures remain essential to effectively combat hookworm disease. In terms of population distribution, women and individuals aged 45 and above are identified as high-risk groups for hookworm infection. These people, primarily involved in agricultural pursuits in rural settings, are particularly vulnerable to infection by hookworm larvae. Previous prevention and control efforts have targeted middle-aged and elderly individuals, as well as women, for the prevention and control of hookworm disease [24–28]. Moving forward, these populations will continue to be a primary focus in subsequent prevention and control initiatives.

This study identified the top 8 influential variables in the RF model, and examined their interdependency with hookworm disease. The analysis highlighted the frequency of barefoot farming as the most significant factor influencing hookworm disease. Both “occasional” and “frequent” barefoot farming were found to be risk factors for hookworm infection, with individuals engaging in frequent barefoot labor facing a fourfold higher risk compared to those not involved in such activities. These findings are in line with previous research [33–37] and also consistent with the known life cycle and transmission pathways of hookworm. The primary mode of hookworm transmission is through skin contact with infective larvae, with barefoot farming increasing the likelihood of exposure to infective larvae in the soil. In the control and prevention of hookworm disease, promoting changes in barefoot farming practices through health education has been a key control strategy. In the context of landform variable, “plateaus”, “mountains”, and “hills” have been identified as risk factors for hookworm disease when compared to “plains”. This finding aligns with results from the second national survey and other studies [38–39]. The underlying reason for this phenomenon is that PLADs with elevated hookworm infection rates—such as Yunnan, Sichuan, Chongqing, and Guizhou—are predominantly situated in regions characterized by plateaus, mountains, and hills. These areas generally exhibit a warm and humid climate with rich vegetation, which creates conducive conditions for the survival and transmission of hookworms. Additionally, some plateau and mountainous regions experience slower economic development relative to plain areas, thereby impeding local efforts in hookworm control [39]. In contrast, PLADs that are more economically developed and exhibit lower hookworm infection rates are frequently located in plain regions. The observed variations in hookworm infection rates associated with changes in longitude can largely be attributed to the disparities in the actual distribution of hookworms across different longitudinal ranges (regions) within China.

Within the longitudinal ranges of less than 100° and greater than 122°, there was no significant variation in hookworm infection rates as longitude changes. This phenomenon can be attributed to the fact that regions with a longitude of less than 100° mainly encompass economically underdeveloped western areas, including Xinjiang, Qinghai, Xizang, Gansu, and Inner Mongolia, with low infection rates of hookworm. Conversely, the region with a longitude greater than 122° primarily includes the north-eastern PLADs of Heilongjiang, Jilin, and Liaoning, where low temperatures and dry climates are unfavorable for the survival of hookworms, resulting in generally low and relatively uniform infection rates. In the longitudinal range of 100° to 113°, hookworm infection rates tend to decrease with increasing longitude. This trend is largely due to the western part of this range (characterized by relatively smaller longitudes), which encompasses most areas of Sichuan and Yunnan, as well as regions in Chongqing, Hainan, and Guizhou, all of which have relatively high hookworm infection rates. As one moves eastward (with increasing longitude), there is a gradual transition to central PLADs characterized by lower hookworm infection rates. Furthermore, within the longitudinal range of 114° to 122°, hookworm infection rates exhibited a slight increase with increasing longitude. This is primarily because the western portion of this range (with relatively smaller longitudes) includes western PLADs with low hookworm infection rates, while moving eastward (with increasing longitude) leads to PLADs such as Zhejiang and Fujian, which have comparatively higher infection rates.

Previous studies have explored the relationship between the distribution of hookworm and climate factors, identifying temperature, humidity, and rainfall as significant influences on the geographical distribution of *Ascaris lumbricoides*, and *Trichuris trichuria*, and hookworm. However, these investigations did not provide an in-depth analysis of the relationship between climate factors and hookworm infection rates [37,38,40,41]. The present study employs a machine learning model to conduct a more comprehensive analysis of the dependency between hookworm disease and climate factors. The findings reveal that the main climate factors influencing hookworm disease include the average relative humidity in the third quarter (Rhu03), monthly average sunlight duration (SSD), and average temperature in the fourth quarter (Tem04). Specifically, when the average relative humidity in the third quarter (Rhu03) was < 73, the hookworm infection rate remained very low and exhibits no significant variation with changes of Rhu03. Conversely, when Rhu03 falls within the range of 73–93, there was a marked increase in the hookworm infection rate as Rhu03 rises. This indicates that extremely low relative humidity is unfavorable for the survival of hookworms, and it is only when relative humidity increases to a certain threshold (73) that conditions become conducive to their survival. Moreover, within a specific range of humidity, higher levels of humidity are associated with increased hookworm infection rates. This conclusion is consistent with the survival habits of hookworm, particularly the larvae, which thrive in relatively high-humidity environments. Suitable humidity facilitate the survival and transmission of hookworm [42]. When the monthly average sunlight duration in the third quarter (Ssd03) was < 130 hours, the hookworm infection rate significantly decreases as Ssd03 increases. However, within the range of 130–300 hours, variations in Ssd03 did not lead to significant changes in the hookworm infection rate. This observation suggests that, within a specific range of sunlight duration, the infection rate of hookworms is inversely related to sunlight exposure; excessive sunlight appears to hinder the growth, reproduction, and transmission of hookworms. When sunlight duration surpasses a certain threshold, it becomes highly unfavorable for hookworm survival, resulting in a marked reduction in infection rates. In the fourth quarter, the average temperature (Tem04) within the range of -30–15°C is associated with extremely low hookworm infection rates, with no significant changes noted as Tem04 varies. Conversely, when Tem04 falls between 15 and 22°C, the hookworm infection rate increases with rising temperatures. This indicates that low temperatures are not conducive to the growth and reproduction of hookworms, and within a defined temperature range, the infection rate is directly proportional to temperature. These findings align with previous research, which indicates that hookworms can survive for over 15 weeks under suitable temperature and humidity conditions, typically becoming active at temperatures between 15 and 18°C, and achieving maximum survival rates at 20–30°C [43]. The hookworm infection rate is primarily influenced by temperature, humidity, and sunlight duration during the third and fourth quarters. It is plausible that elevated temperatures in the third quarter lead to increased instances of residents working barefoot in an effort to cool off. Furthermore, the conclusion of the third quarter and the onset of the fourth quarter coincide with the harvest season, during which residents spend prolonged periods working in the fields. These factors likely contribute to increased exposure to hookworm larvae, resulting in a higher infection rate of hookworm disease.

In this research, the development of a machine learning model focused on the risk factors of hookworm disease has highlighted the substantial impact of climatic variables (Rhu03, Ssd, and Tem04) and key hygiene-related behaviors (such as barefoot farming) on the infection rate of hookworm disease. The influence of climate factors on hookworm infections can serve as a predictive tool for assessing the potential epidemic intensity of hookworm disease across various natural environments. Furthermore, the notable impact of barefoot labor practices on the prevalence of hookworm disease underscores the necessity for future prevention and control strategies to prioritize enhancing public awareness of hygiene knowledge and promoting adherence to hygienic behaviors. Additionally, it is essential to address and modify the critical hygiene behavior associated with barefoot labor.

Among the 23 variables included in this study, significant multicollinearity was observed among certain climatic factors. Although traditional statistical models are sensitive to highly collinear variables, which may compromise model stability, prior research has demonstrated that machine learning models exhibit greater tolerance to multicollinearity due to their inherent algorithmic properties. Through RFE, the optimal variable subset was identified, which retained several strongly correlated variables. Nevertheless, variable importance assessment effectively differentiated the relative contributions of each predictor to the dependent variable, thereby mitigating the impact of multicollinearity and reducing data noise introduced by redundant variables. Consequently, the presence of multicollinearity in the selected variable subset did not significantly impair the predictive performance of the machine learning models constructed in this study [44].

## Conclusions

Based on the analysis of the 2016–2021 national surveillance data, this research highlighted the impact of climate factors and hygiene behavior on hookworm infection rates in China. This research delineates the distinct spatial clustering of hookworm disease in China, with persistent hotspots concentrated in the south-western region, particularly affecting women and elderly populations. Machine learning modeling identified barefoot farming as a key behavioral risk factor, and revealed significant positive correlations between infection rates and temperature/humidity, and negative associations with sunlight exposure - with these climatic factors being most pronounced during the third and fourth quarters. To advance transmission control and elimination efforts, it is recommend to establish targeted intervention demonstration zones in high-endemicity areas, implementing gender- and age-specific prevention programs, such as target-population screening, and targeted health education during special transmission seasons, thereby adopting a “core-area-focused, population-specific” strategy to drive nationwide hookworm control.

## Supporting information

S1 File Collinearity analysis between meteorological factors. (PDF)

S2 File Collinearity analysis between environmental and anthropogenic factors. (PDF)

S1 Data EVP_relevant data.zip. (ZIP)

S2 Data PRE_relevant data.zip. (ZIP)

S3 Data RHU_relevant data.zip. (ZIP)

S4 Data SSD_relevant data.zip. (ZIP)

S5 Data TEM_relevant data.zip. (ZIP)
