# Supplementary figures and images for: Spatial distribution patterns and risk factors of hookworm disease in China: A study based on successive national surveillance
Source: PLoS Negl Trop Dis. 2025 Sep 30;19(9):e0013526. doi: 10.1371/journal.pntd.0013526 (PMC12483247; doi:10.1371/journal.pntd.0013526)

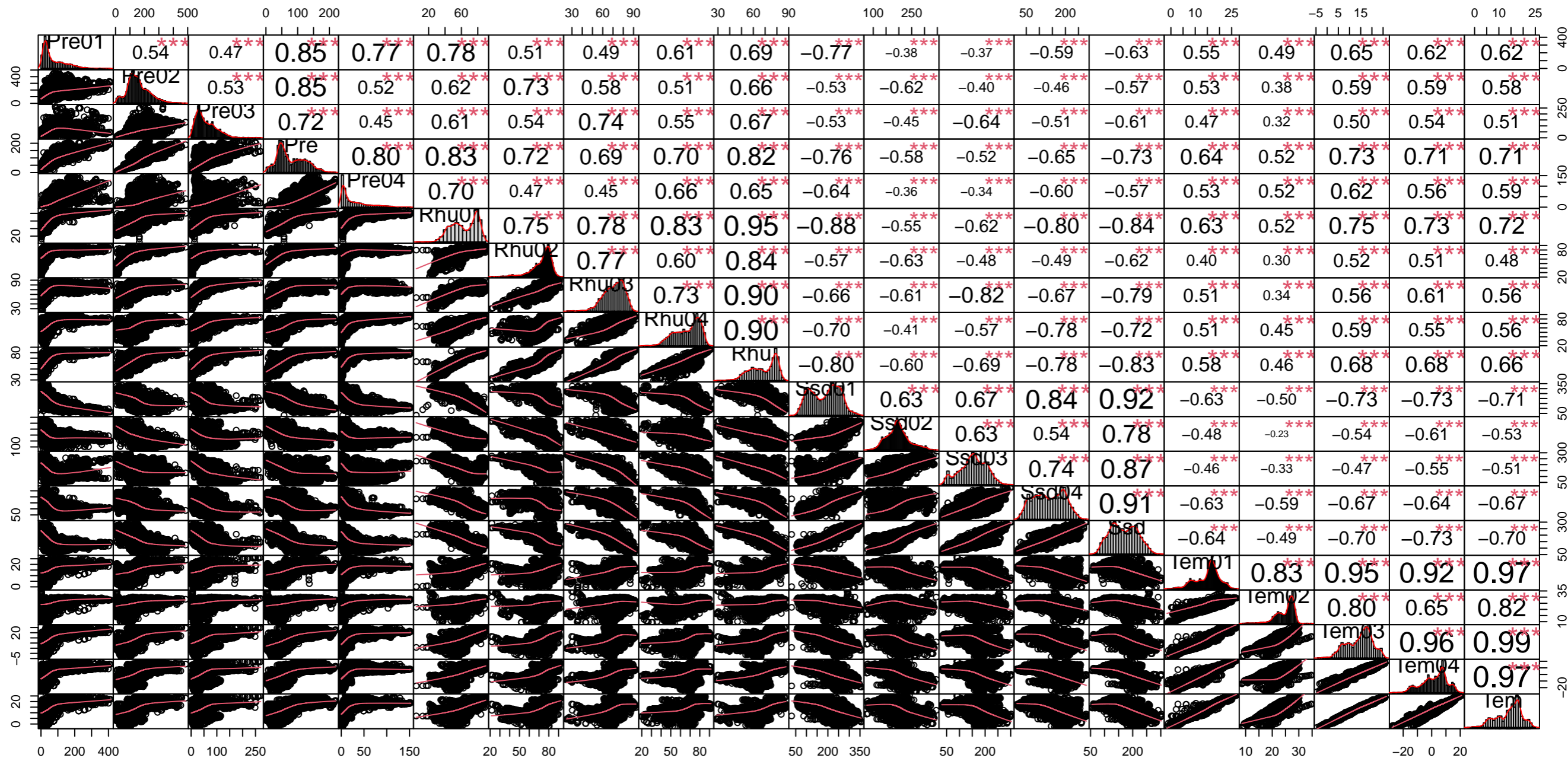

Supplement: S1 File — (PDF) [file pntd.0013526.s001.pdf]

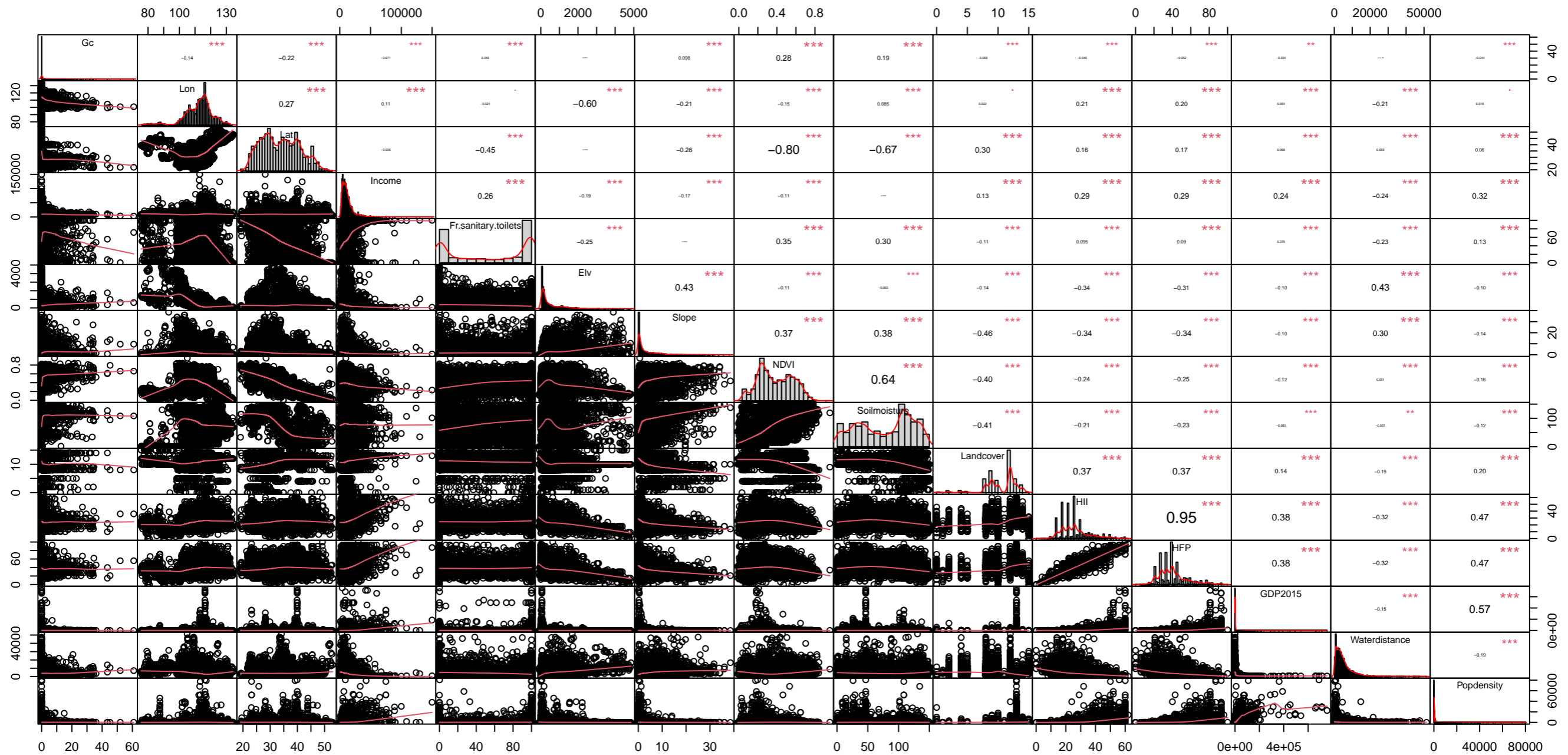

Supplement: S2 File — (PDF) [file pntd.0013526.s002.pdf]
